# 

**Published:** 1920-12-25

**Authors:** 


### Matrons' and Sisters' Appointments.

MATERNITY HOSPITAL, NELSON.—Miss Ellen Barker has been appointed matron. She was trained at the St. Pancras Infirmary and the Woolwich Military Families' Hospital, and later held successively the posts of staff midwife at the Jessop Hospital, Sheffield, ward sister at the Sheffield Children's Hospital, matron of special maternity hospital under the Bradford Corporation, and superintendent of district midwives.

COTTAGE HOSPITAL, LLANDUDNO.—Miss Myfanwy Eurgain has been appointed sister. She was trained at the Liverpool Royal Infirmary.

TAUNTON AND SOMERSET HOSPITAL.—Miss Ella Spackman has been appointed night superintendent. She was trained at St. Bartholomew's Hospital, has been ward sister and night superintendent at the Norfolk War Hospital, and charge sister in the Q.A.I.M.N.S.R. in Salonika.

FEVER HOSPITAL, BIRKENHEAD.—Miss Winnie Davies has been appointed sister. She was trained at Inverness Infirmary and Ladywell Sanatorium, Manchester.

ENHAM VILLAGE CENTRE FOR DISABLED EX-SERVICE MEN, ANDOVER.—Miss A. M. Logan has been appointed assistant matron. She was trained at the Sunderland Royal Infirmary, where she was later ward sister and theatre sister; during the war she served as sister in the Royal Naval Nursing Service. Miss Logan is at present taking a special housekeeping course at the General Hospital, Nottingham.

WELLHOUSE HOSPITAL, BARNET.—Miss B. Sabey and Miss M. Coulmare have been appointed ward sisters. Miss Sabey was trained at Northampton General Hospital, later becoming sister there. Miss Coulmare was trained at the London Hospital, and has been home sister at the Salisbury Infirmary.

ROMFORD UNION INFIRMARY.—Miss Grace Elsie Page has been appointed charge sister. She was trained at the Kensington Infirmary, where she was later ward sister, which post she has held also at Norwich and Chesterfield Infirmaries.

DR. CYRIL M. PROBERT, of Mountain Ash, has been appointed assistant medical officer at the Merthyr Infirmary and Pontsarm Sanatorium. A Guy's man, where he was surgical dresser to Sir Alfred Fripp, Dr. Probert served as a surgeon sub-lieutenant in the Navy during the war.

